# Whey Proteins as a Potential Co-Surfactant with *Aesculus hippocastanum* L. as a Stabilizer in Nanoemulsions Derived from Hempseed Oil

**DOI:** 10.3390/molecules26195856

**Published:** 2021-09-27

**Authors:** Wojciech Smułek, Przemysław Siejak, Farahnaz Fathordoobady, Łukasz Masewicz, Yigong Guo, Małgorzata Jarzębska, David D. Kitts, Przemysław Łukasz Kowalczewski, Hanna Maria Baranowska, Jerzy Stangierski, Anna Szwajca, Anubhav Pratap-Singh, Maciej Jarzębski

**Affiliations:** 1 Institute of Chemical Technology and Engineering, Poznan University of Technology, Berdychowo 4, 60-695 Poznań, Poland; wojciech.smulek@put.poznan.pl; 2 Department of Physics and Biophysics, Faculty of Food Science and Nutrition, Poznań University of Life Sciences, Wojska Polskiego 38/42, 60-637 Poznań, Poland; przemyslaw.siejak@up.poznan.pl (P.S.); lukasz.masewicz@up.poznan.pl (Ł.M.); hanna.baranowska@up.poznan.pl (H.M.B.); 3 Food, Nutrition and Health Program, Faculty of Land & Food Systems, The University of British Columbia, 2205 East Mall, Vancouver, BC V6T 1Z4, Canada; farah.fathordoobady@ubc.ca (F.F.); yigong.guo@ubc.ca (Y.G.); david.kitts@ubc.ca (D.D.K.); 4 Independent Researcher, 60-343 Poznań, Poland; malgorzata.jarzebska@o2.pl; 5 Department of Food Technology of Plant Origin, Poznań University of Life Sciences, 31 Wojska Polskiego St., 60-624 Poznań, Poland; przemyslaw.kowalczewski@up.poznan.pl; 6 Department of Food Quality and Safety Management, Faculty of Food Science and Nutrition, Poznań University of Life Sciences, Wojska Polskiego 31/33, 60-624 Poznań, Poland; jerzy.stangierski@up.poznan.pl; 7 Department of Synthesis and Structure of Organic Compounds, Faculty of Chemistry, Adam Mickiewicz University in Poznań, Uniwersytetu Poznańskiego 8, 61-614 Poznań, Poland; anna.szwajca@amu.edu.pl

**Keywords:** nanoemulsion, whey protein, *Aesculus hippocastanum* L., hempseed oil, emulsion stability, droplet size

## Abstract

The use of natural surfactants including plant extracts, plant hydrocolloids and proteins in nanoemulsion systems has received commercial interest due to demonstrated safety of use and potential health benefits of plant products. In this study, a whey protein isolate (WPI) from a byproduct of cheese production was used to stabilize a nanoemulsion formulation that contained hempseed oil and the *Aesculus hippocastanum* L. extract (AHE). A Box–Behnken experimental design was used to set the formulation criteria and the optimal nanoemulsion conditions, used subsequently in follow-up experiments that measured specifically emulsion droplet size distribution, stability tests and visual quality. Regression analysis showed that the concentration of HSO and the interaction between HSO and the WPI were the most significant factors affecting the emulsion polydispersity index and droplet size (nm) (*p* < 0.05). Rheological tests, Fourier transform infrared spectroscopy (FTIR) analysis and L*a*b* color parameters were also taken to characterize the physicochemical properties of the emulsions. Emulsion systems with a higher concentration of the AHE had a potential metabolic activity up to 84% in a microbiological assay. It can be concluded from our results that the nanoemulsion system described herein is a safe and stable formulation with potential biological activity and health benefits that complement its use in the food industry.

## 1. Introduction

Emulsions are widely used in many industries and have an important role in the food industry to stabilize many different formulations that have either oil-in-water (O/W) or water-in-oil (W/O) dispersions [1]. Stability of emulsions is influenced by many factors, e.g., the concentration and ratio of individual phases, methods of preparation, storage condition, as well as the presence of compounds demonstrating the surface-active property (stabilizers) [2]. Therefore, new sources of natural, effective stabilizers, or surfactants, are in high demand to use plant-based ingredients that provide bioactive extracts [3,4,5], hydrocolloid activity [6], plant proteins [7,8,9]. Many biopolymers (proteins, polysaccharides or the combinations) that are used to assist in the formation of an emulsion have excellent effectiveness since they contain both hydrophilic and hydrophobic groups capable of lowering the surface tension between water and the oil phase [10]. Such proteins as collagen, whey protein, soy protein isolate and casein are the most commonly used due to a high emulsifying index and the water/oil holding capacity [11].

Hempseed oil (HSO), derived from the seeds of *Cannabis sativa* L., is recognized for both nutritional, health-promoting and bioactive properties [12,13]. It is a good source of both n-3 and n-6 essential fatty acids, specifically linoleic acid (55%) and alpha-linolenic acid (20%), with concentrations relatively higher than in other vegetable oils [14]. HSO is also a source of gamma linoleic acid (GLA) not found in many vegetable oils, but with noted bioactive properties. HSO’s health advantages have been ascribed to the 3:1 n-3/n-6 fatty acid ratio, which is an established optimum for human nutrition uptake [14], and the presence of GLA [15]. Additionally, the presence of minor components, such as tocopherol, tocotrienols, carotenes, minerals, terpenoids and β-sitosterol also add to the nutritional value of HSO. Besides these health effects, HSO is often used in formulations that require high miscibility with water [16], which reduces the reliance of additional surfactants for use in nanoemulsions. Accordingly, HSO has been used in cosmetic, nutraceutical, food and functional food industries.

Dairy proteins have been widely used as emulsifiers in food industry. They adsorb to the oil droplet interface, producing a consistent protective film that can help prevent droplet aggregation [17]. They have also been used as emulsifiers in nanoemulsion formulations. Whey protein (WP), with a noted high nutritional value, is often used as an ingredient in many food products and for the production of sports nutrition [18,19]. Whey proteins are a good source of essential amino acids, and globular proteins isolated from whey are noted for having biological activities: antioxidant, immunostimulatory and anti-obesity [20]. They are also effective emulsifiers in food formulations because their amphiphilic properties enable relatively stable gels and microcapsules without additional chemical additives. For example, WP has been used to encapsulate probiotics [21,22,23]. Iqbal et al. [24] used the WI in the formation to generate a three-dimensional network in an oil/water phase. However, the resulting emulsions did not exhibit ideal plastic behavior. Since WP can be prepared as an isolate or a concentrate, the WPI has been used to stabilize nanoemulsion formulations containing peanut oil, corn oil, β-carotene in sunflower oil and α-Tocopherol in palm oil. It was found that the lowest degradation of β-carotene at 55 °C occurred when WPI was used as stabilizer in the nanoemulsion system. This was probably related to the substantial antioxidant property of WPI [25].

*Aesculus hippocastanum* L. is also known as horse chestnut [26] and contains many biologically active compounds including escin, a mixture of saponins with medicinal properties. The health beneficial effects of AH include anti-inflammatory and anti-edematous [27] properties, with potential benefits for kidney disease in diabetic nephropathy [28]. Due to the high content of polyphenols, such as quercetin and kaempferol [29], *Aesculus hippocastanum* L. bark also exhibits antioxidant and antitumor activities [30]. In addition, the extracts from *Aesculus hippocastanum* L. (AHE) with a high content of saponins [31] exhibited high interfacial activity due to hydrophobic aglycone and hydrophilic sugar residues [12]. The United States Department of Agriculture (USDA) considers the AHE to be generally regarded as safe, and the European Union has also approved it for use as a foam stabilizer in beverages [12]. AHE was found to be an effective stabilizer when used as a stabilizer in hempseed oil (HSO) nanoemulsions [12]. HSO–nanoemulsion formulations containing 2 g/L AHE also produce smaller droplets.

The purpose of this study was to prepare an O/W formulation of an HSO nanoemulsion system that was stabilized with an optimal concentration of the WPI and the AHE. The goal was to obtain both acceptable stability of the emulsion and also potential biological activity of its ingredients for both food and biomedical applications. We also focused on using the WPI as a potential cosurfactant.

## 2. Results and Discussion

### 2.1. Stability Tests

**Figure 1:**
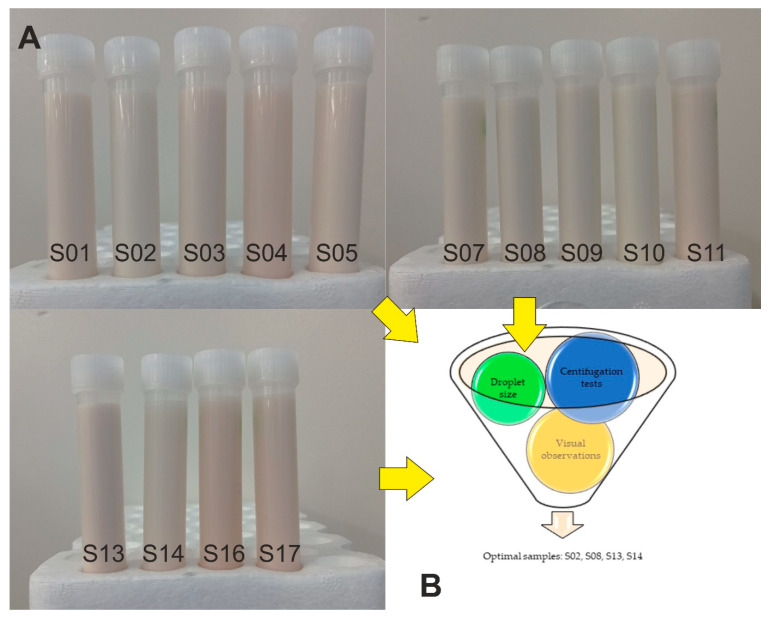
(**A**) The emulsion samples after 24 h. (**B**) Criteria of selecting the optimal samples composition for further studies.

Three level Box–Behnken experiment design with response surface methodology was applied for the experiment setup (Table 1). To evaluate the efficiency of the emulsion preparation, a two-step homogenization process using both a high-speed homogenizer and an ultrasound homogenizer was used. To compare the behavior of the emulsion composition, we determined the emulsification index (EI) [31] which measured the emulsified layer relative to the total volume of the mixture. The EI results were compared with visual observation, which are presented in Figure 1 and show samples 24 h after homogenization. The visual measures confirmed that the two-step preparation process strongly increased the homogeneity of the emulsion in contrast to the one-step process [12,30]. One day after the preparation, no visible differences in samples were produced, and homogeneity was noted within the total volume. Visual observation confirmed a slight color difference in the samples, which were attributed to the different HSO and AHE concentrations, respectively.

We employed additional centrifugation tests to accelerate evaluation of the sample emulsion. The ratio of the optical density (measured at 600 nm) of the samples before and after centrifugation was used as an indicator for emulsion stability. The results were collected and presented in Table 2. A simple correlation between the concentration of individual emulsion components on emulsion stability after centrifugation could not be established. Only samples with the lowest HSO content (i.e., 1%) were characterized by higher stability. The highest values for this parameter (>50%) were also observed for samples S16 and S17, respectively, with the same concentration of HSO (1%). These results suggest that greater whey concentrations promoted the formation of relatively more stable emulsions.

### 2.2. Droplet Size Studies

Droplet size and PDI (polydisperisty index) are key factors affecting emulsion stability. Based on the experimental design used for preliminary studies (Table 3), different concentrations of HSO, AHE and whey protein were used to find the optimal droplet size distribution of nanoemulsion formulations. According to our previous studies [9,32], we decided to eliminate runs S06, S12 and S15 from the experimental design model (Table 1) for the evaluation of the droplet size. Analysis of the results presented in Table 3 for the center points of the Box–Behnken design were performed on samples S01, S05, S06, S12, S15, and similar results for Z-ave, PDI, average peak maximum by intensity were recorded for the S01 and S05 samples (see the standard deviation range of three replications). 

In contrast to previous studies [33,34], we determined the droplet size of the emulsion systems as delivered after preparing the emulsion without additional dilution since dilution may affect the emulsion stability. Furthermore, based on the principles of the dynamic light scattering (DLS) method used herein and apparatus limitations, we chose to round droplet size numbers up to a full number. Moreover, based on previous experience from studies with different high-PDI systems [35], we considered droplet/particle size by intensity and number. Detailed studies of droplet size distributions clearly showed that even a small fraction of larger particles/droplets or accidental contaminations impacted the Z-ave and size distribution. Results presented in Table 2 exhibit that the major percentage of droplets were much smaller than average size distribution by intensity peak maximum. For example, the average maximum of the peak by number for sample S08 was 23 nm; however, its light scattering intensity was 318 nm.

According to the regression analysis and the ANOVA, we were able to predict Z-Ave for samples using a quadratic model (*p* < 0.05). The relative significance in order of individual factors according to the greatest effect was determined to be as follows: HSO (%) > whey (%) > AHE. The interaction of HSO and whey was a significant factor affecting Z-ave of samples (*p* < 0.05). The significant interaction effect of HSO and a poloxamer used as a surfactant to produce an HSO-based nanoemulsion was previously reported [36]. The ANOVA and the regression analysis also showed that the PDI of nanoemulsion samples could be fit to a linear model with HSO (%) as the only significant factor (*p* < 0.05). To find an optimal and stable formulation and further possible applications of the emulsion system, other characterizations such as droplet size distribution, visual, centrifugation and microscopic properties of the emulsion need to be considered in addition to Z-ave and the PDI. Additional stability studies, such as identification of destabilization mechanisms including creaming, sedimentation, flocculation, coalescence can also confirm the stability potential of an emulsion system. Based on the preliminary stability tests as well as droplet size distribution, we selected four samples for further analysis (Figure 1B).

**Figure 2:**
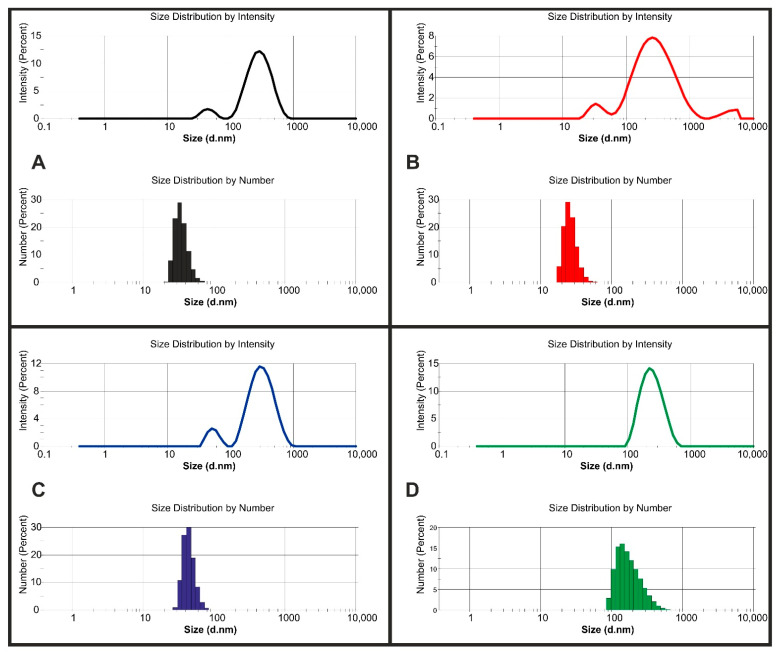
Droplet size distribution by intensity and by number: (**A**) S02, (**B**) S08, (**C**) S13, (**D**) S14.

Figure 2 presents detailed analysis of droplet size distribution from four selected samples. All the selected samples contained a whey concentration of 4, except for S14, which had a whey concentration of 2.5%. According to the DLS results of S14 (Figure 2D), the scattered light intensity and droplet size by number were not different. This result was confirmed by a low PDI value (0.190). Microscopic observation of sample S14 (Figure 2D) also confirmed homogeneity of the samples. In addition, in comparison with other samples, single droplets were easy to distinguish at low magnification (e.g., ×40).

**Figure 3:**
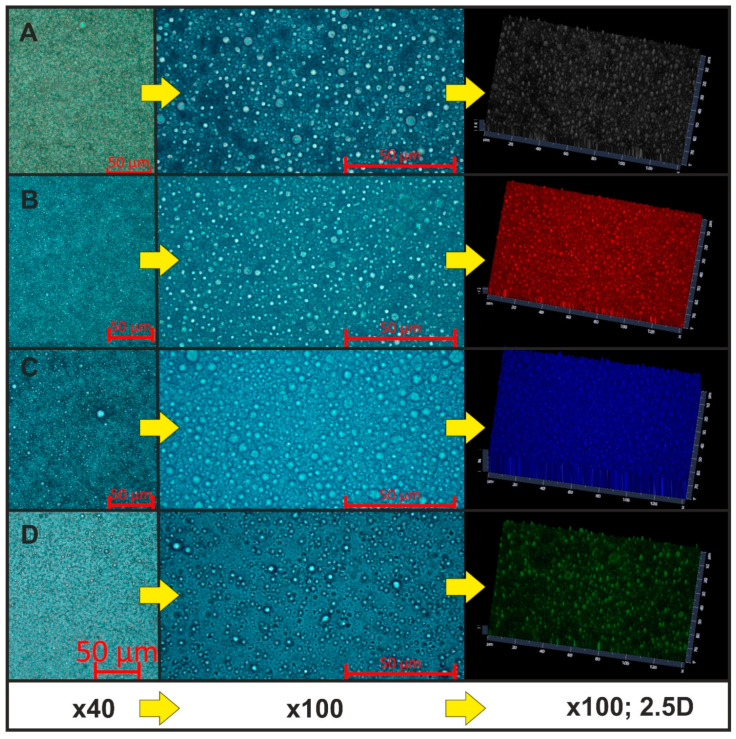
Microscopic images of the emulsion droplets (magnification, ×40, ×100, and ×100; 2.5D image): (**A**) S02, (**B**) S08, (**C**) S13, (**D**) S14 (the images were adjusted as the best fit and colored with software).

Based on our previous observations for the HSO emulsion system stabilized by the AHE [12], we decided to evaluate emulsion droplet distribution and sample homogeneity using light microscopy with an inverted microscope. To prevent fast evaporation of samples, the emulsions were inserted in a corvette equipped with slide channels. The images presented in Figure 3 were taken a few minutes after the emulsion injection. This method prevents the flow of injected liquid artefacts. For unstable systems, a short break in time enabled the observed coalescence of the droplets. With lower magnification, high homogeneity of samples S02, S08 and S13 was observed (Figure 3A–C). Only a small fraction of larger droplets could be distinguished. This observation corresponded with the DLS results in case of droplet size distribution by intensity, where a more intensive signal was detected for the samples with larger hydrodynamic diameters (Figure 2A–C). The presence of the smaller droplets fraction determined by DLS was probably caused by two-step processing using ultrasound treatment for preparing this emulsion. Some of the studies suggested that ultrasound treatment as well as ultrahigh-pressure homogenization caused an increase in the α-helix structure content of whey protein and a decrease in the β-sheet component of whey protein [37,38].

Microscopic imaging for nanoemulsion systems with proteins presented by Zhu et al. [39] corresponded to our findings. In their studies, optical microscopic imaging was compared with confocal laser scanning microscopy (CLSM). It was suggested that using CLSM allowed for the determination of a core–shell structure in the emulsion systems where proteins were located at the surface of the emulsion droplets. More detailed studies are needed for our nanoemulsion systems. First of all, signals from all the fluorescent structures should be identified and separated (i.e., from HSO, AHE) [12,31]. Then, additional studies on the impact of whey protein on the fluorescent behavior of the components should be investigated. Ren and Giusti [40] showed that anthocyanin-rich extracts decreased the fluorescence intensity of whey protein while increasing λ_max_. The study concluded that thermally induced whey protein was effective in protecting anthocyanin from color degradation. Using an optical microscope, we focused only on the verification of the homogeneity of the samples as well as possible coalescence (which was not observed in the optimal nanoemulsions). Nevertheless, we strongly recommend using more than one technique for the analysis of the emulsion systems droplets size.

### 2.3. Rheological Tests

**Figure 4:**
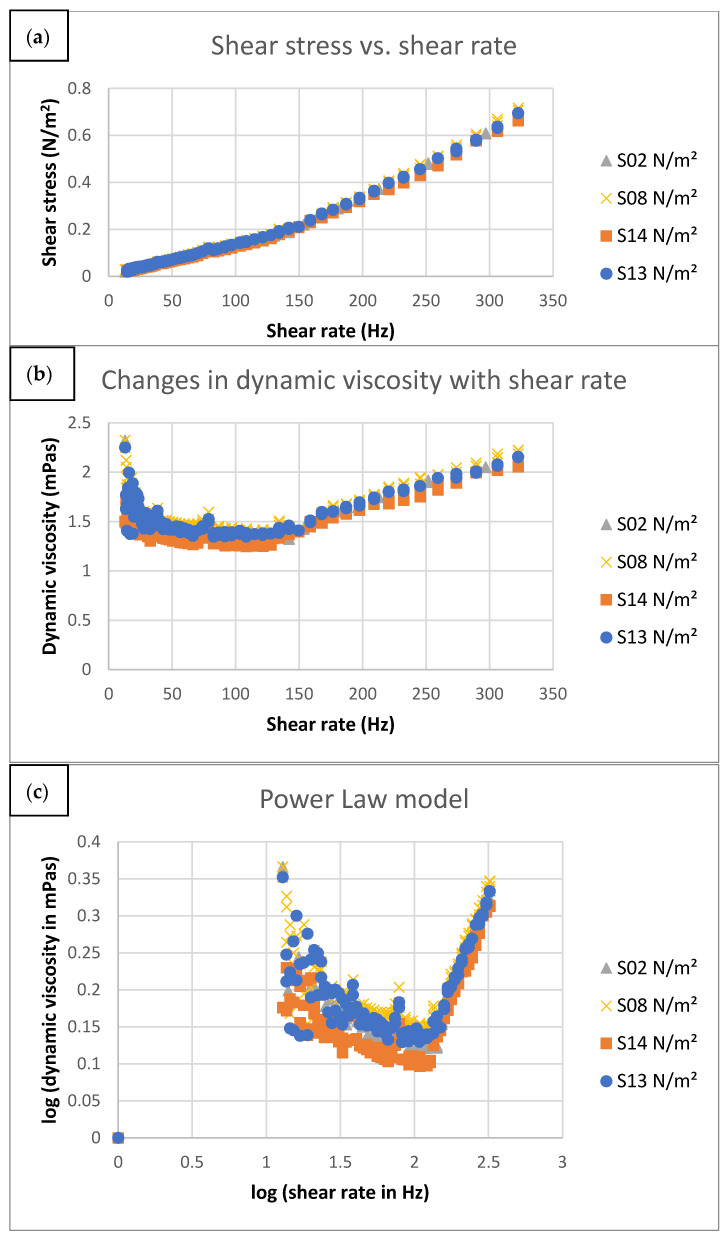
Rheological test results of the S02, S08, S13, and S14 samples: (**a**) flow curve, (**b**) dynamic viscosity changes, (**c**) power law model.

Figure 4a shows the flow curves of the various samples, which illustrate the samples’ rheological properties. The flow curves showed a non-Newtonian behavior, with a decreasing slope (viscosity) up to a certain cut-off, suggesting typical pseudoplastic behavior. However, the viscosity increased after a certain shear rate, which is a typical property of a dilatant fluid. The changes in the dynamic viscosity of the samples with increasing shear rate are shown in Figure 4b. It was apparent that the S08 sample had the highest viscosity, followed by S13 and S02, with S14 being least viscous at various shear rates. All the samples exhibited two types of behavior: first, shear thinning, and then shear thickening, after a particular shear rate cut-off. This became more apparent in Figure 4c when the power law was used to model these curves to evaluate the coefficient that depicted clearly the two zones, with a cut-off close to 130 Hz. As a result, a broken power law model (Equation (1)) was used to describe the flow behavior of the samples.

(1) $$\eta =K_{1}{\left(\dot{\gamma }\right)}^{n_{1}-1}\;\mathrm{for}\;\dot{\gamma }<130\;=K_{2}{\left(\dot{\gamma }\right)}^{n_{2}-1}\;\mathrm{for}\;\dot{\gamma }\geq 130$$

where η is the dynamic viscosity, K_1_ and K_2_ are the consistency coefficients, $\dot{\gamma }$ is the shear rate and n_1_ and n_2_ are the flow behavior indices.

Table 4 shows the broken power law model parameters. The consistency coefficient K_1_ at shear rates less than 130 Hz was found to be highest for S08, while those of other samples were not significantly different. This measure is an indicator of the initial system viscosity [41], suggesting that sample S08 had higher viscosity to begin with, which was sustained even at changing shear rates. 

The higher viscosity of S08 as compared to the other samples could be attributed to the highest HSO (5%) content amongst the four tested samples. Furthermore, S14 was found to possess the lowest viscosity on account of having the lowest HSO content (1%). This showed that a higher oil loading was associated with higher viscosity, which is consistent with observations of other researchers [42,43,44]. Rha [45] and Jarzebski et al. [12] attributed this phenomenon of increasing viscosity with increasing oil loading to the greater formation of interphase layers, creating a larger barrier between the emulsion components. 

At a shear rate of 130 Hz, all the samples exhibited a transition from shear thinning behavior to shear thickening behavior. Again, samples S08, having the highest oil loading, and sample S14, with the lowest oil and whey loading, demonstrated the highest and the lowest viscosity, respectively. This transition from shear thinning to the shear thickening behavior could be attributed to the phenomenon that at very high shear rates, tremendous turbulence occurs. When such turbulence occurs after a certain shear rate cut-off (which was 130 Hz in our case), any increase in the shear rate will result in increased turbulence, resulting in increased viscous dissipation and higher resistance to flow, which in turn makes the flow appear as shear-thickening. This effect is a typical example of the Taylor vortex flow in shear-thinning fluids [46,47]. The interpretation of Chhabra and Richardson [48] could be used to explain such transitionary behavior of our shear-thinning samples. At rest, the emulsion has sufficient interfacial tension to be stable. At low shear rates, lubrication for particle motion of the continuous phase between the plates is provided by the dispersed oil phase resulting in decreased stress with increasing shear (shear-thinning behavior). However, at high shear rates, the emulsion breaks, and the dispersed phase is completely separated from the continuous phase due to centrifugal forces. Furthermore, continuous and dispersed phases expand or dilate slightly under increasing shear strain, resulting in increased friction and shear stress, causing the dynamic viscosity to increase rapidly with shear rate.

### 2.4. FTIR

**Figure 5:**
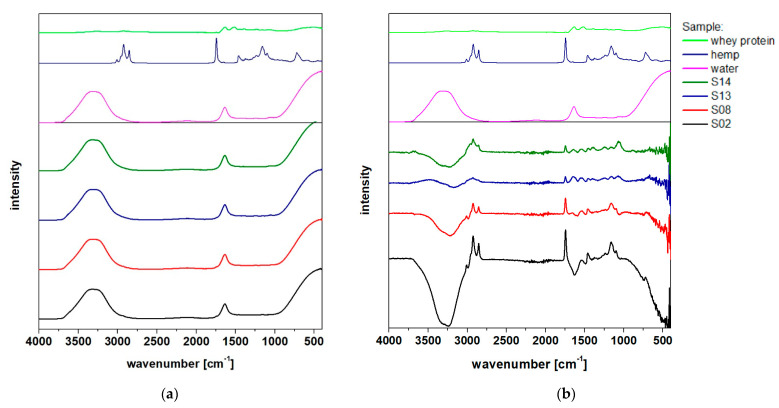
FTIR spectra of investigated samples with (**a**) air or (**b**) water as the background.

The FTIR spectra were recorded with air or water as the background. Both series of results are presented in Figure 5.

The HSO spectrum shows clear signals originating from the C–H bonds (around 2950 cm^−1^) and C = O (at 1700 cm^−1^), respectively. The absence of signals that are characteristic of hydroxyl group bonds proves that there were no significant amounts of free fatty acids and hydroxylated acids in the oil. A relatively weak but distinctive signal slightly above 3000 cm^−1^ confirms the existence of double bonds between carbon atoms, which most likely originated from unsaturated fatty acid residues in HSO, as confirmed by others [49]. 

Among the signals common to all emulsion samples, those arising from the O–H bonds (at about 3250 cm^−1^), aliphatic C–H (at 2800–2950 cm^−1^) and C = O (at 1700 cm^−1^) should be distinguished. Although the differences in the intensities of the individual bands can be explained by the different composition of the emulsions, it is important to note that these differences were not only in intensity, but also in the position of the signals in the 1050–1150 cm^−1^ range. They could originate from vibrations of the C–O bonds (to a lesser extent, of C–N, which occurs in the protein structure). A similar shift, visible as a change in shape, is present for vibrations from the O–H group. This shift may indicate a variation in the strength and configuration of the hydrogen bonds between polar components of the tested emulsions, suggesting the complex interactions between the emulsion components, which may then have impact on their physicochemical properties [50,51] and plausible biological activity of the samples [52,53].

### 2.5. Color and Refractive Index Analysis

**Figure 6:**
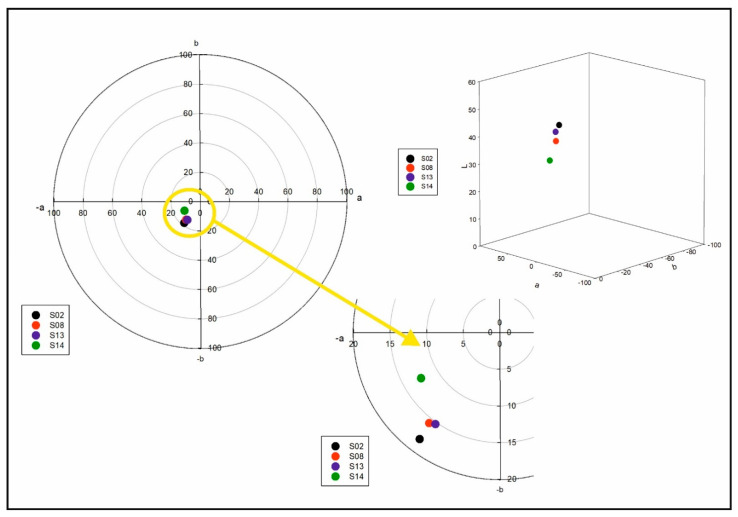
L*a*b* color analysis results of the samples S02, S08, S13, S14.

The results of the L*a*b* color analysis of samples in Figure 6 showed that the S02 sample represented more lightness, followed by S13, S08 and S14. Considering the ratio of ingredients in samples S02, S13 and S08, it was determined that the L* value decreased with higher concentrations of HSO or the AHE, respectively. However, sample S14 having a low content of HSO (1%) and the AHE (1%) featured a low L* value. We hypothesize that other factors might affect the lightness of samples in addition to the concentration of ingredients. Based on droplet size distribution studies, the PDI and Z-ave of S14 were lower compared to other samples (Table 1). In addition, according to McClements and Demetriades (1998), interactions between the ingredients within the emulsion and some other factors such as transmission, reflection, scattering and absorption can also affect the color and appearance of the emulsion [54].

It was also found that the difference between the a* values was negligible. In this analysis, sample S14 showed lower b* values. This observation may also relate to the higher ratio of whey to HSO in this formulation, which caused a shift in favor of a yellow shade. To monitor the color of the emulsions with a unique index, the WI values of the samples were calculated based on the L*, a* and b* values. The WI values were found to be 42.68, 35.86, 40.00 and 29.87 for S02, S08, S13 and S14, respectively, indicating marked difference between sample appearance.

The refractive index recorded for the dispersed phase represented the average refractive indices of individual droplets (Table 5). The theory that the thickness of the phase interface is small enough comparative to the wavelength of the related light would indicate that the interface of droplets plus the oil phase act as an individual dispersed phase [55].

The total appearance of an emulsion can be determined by both light scattering and absorption. Scattering is mainly accountable by recording the turbidity or lightness of an emulsion, while absorption determines the chromatic properties (redness, blueness, greenness, etc.) [56]. The relationship between the color of an emulsion and the refractive index ratio has an important role in foods containing high proportions of the aqueous phase. The refractive index of the samples (Table 4) was similar to the RI of the dispersed phase (water). This property allowed us to use an optical transmission method to analyze emulsions. The level of lipid oxidation in an emulsion, for example, can be determined by adding some chemicals (such as glycerol) to adjust the RI to 1.0, then measuring the spectra of the absorption [57].

### 2.6. Biological Activity

**Figure 7:**
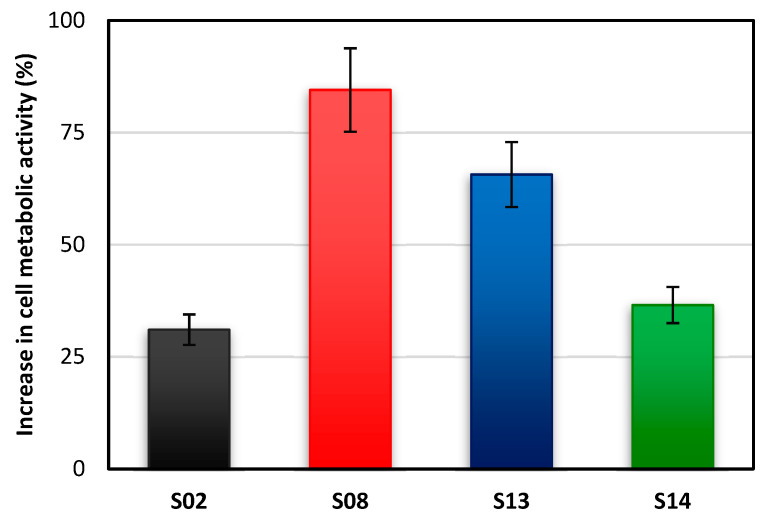
Changes in the metabolic activity of the *Lactobacillus* sp. 2675 cells exposed for 24 h to emulsion samples.

To assess the microbiological safety of optimized emulsions, we determined the effect of emulsions on the *Lactobacillus* sp. 2675 strain, a common probiotic (Figure 7). As a reference sample, cultures without an emulsion were considered (with 0% changes in cell metabolic activity). The highest increase in the bacterial cell metabolic activity (84%) was noticed for sample S08. The observed increase for sample S13 was relatively lower (66%). The increase in metabolic activities were lowest for samples S14 and S02 (37% and 31%, respectively). It can be assumed that higher concentrations of the AHE promoted higher probiotic bacteria growth; however, the small number of experiments needs further confirmation of results. 

Nevertheless, the critical observations were that all samples showed a positive effect on the metabolic activity of *Lactobaccilus* sp. 2675, indication non-toxic properties of the emulsions. Our results correspond directly with results presented by Gharehcheshmeh et al. [58], who reported no effect of a sweet almond and sesame oil nanoemulsions on the growth of *L. delbrueckii* subsp. *bulgaricus*. There are however, few studies showing an effect of AHE on probiotic bacteria. However, former studies showing an impact on probiotic bacteria of polyphenol-rich extracts from blueberry [57,58] or apples [59] agree with our results. 

Nevertheless, the crucial observations are that all the samples exhibited a positive effect on the metabolic activity, which proves the nontoxic properties of the emulsions. Our results correspond directly with the results presented by Gharehcheshmeh et al. who studied the impact of sweet almond and sesame oil nanoemulsions on the growth of *L. delbrueckii* subsp. *bulgaricus*. They did not observe any inhibitory effect of nanoemulsions. 

It is worth adding that the blueberry (*Vaccinium corymbosum*) extract [59,60] also showed beneficial effects on probiotic microorganisms due to the presence of polyphenols, which is also a distinguishing feature of the AHE we used [31]. It should also be noted that plant-derived substances can become an additional nutrient for probiotic microorganisms while providing a protective substance against the adverse effects of the external environment. Ahmad et al. [61] obtained promising results using polyphenols extracted from apples to protect the *Bifidobacterium lactis* bacteria during freezing. In the light of these studies, we can see the great potential of our obtained emulsions to stimulate growth and protect probiotic bacteria.

### 2.7. Water Activity

**Figure 8:**
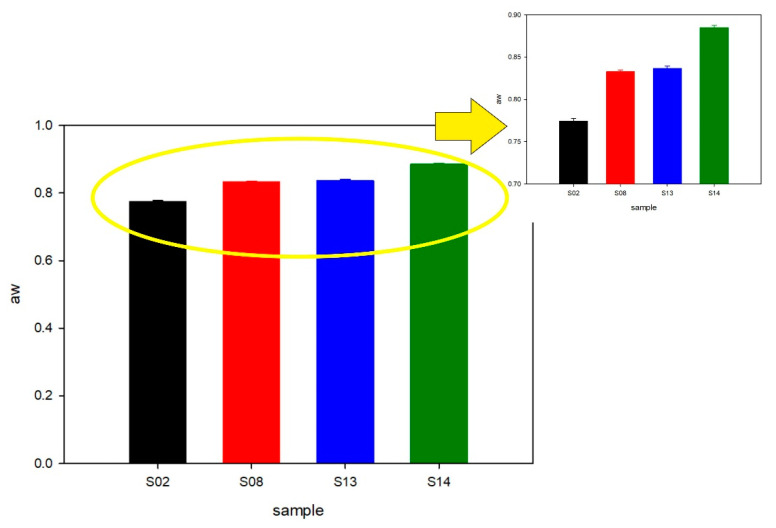
Water activity (aw) results.

Finally, water activity (aw) measurements were taken for the optimized samples (see Figure 8). The lowest water activity was determined in sample S02, where the amount of HSO and the WPI were the highest, but the concentration of the AHE surfactant was the lowest. On the other hand, for the emulsion with the lowest concentration of HSO, the WPI and the AHE (S14) produced the highest water activity. This effect on water activity could have been the result of better uniformity of emulsion droplets and the low concentration of whey. Very similar values of water activity were obtained for the samples with the same concentration of whey, but varied with HSO and the AHE (S08 and S13). Nevertheless, based on the microbiological activity results and visual observation of the samples after two-week storage at room temperature (Figure 1), we concluded that water activity of the samples should be monitored regularly.

## 3. Materials and Methods

### 3.1. Materials

For all the experiments performed in this study, the chemicals used were of analytical grade. The solvents and reagents were purchased from Sigma-Aldrich (Poznań, Poland). Hempseed oil (HSO) was purchased from Złoto Polskie (Kalisz, Poland). Whey protein (Isolac^®^ Instant 125H) was purchased from Carbery Group (Ballineen, Ireland). The plant material, *Aesculus hippocastanum* L. bark, was obtained from Flos (Mokrsko, Poland), and was used to prepare a saponin-rich extract as described [12,31]. The total saponin content in the extract, which was equal to 4 ± 1%, was determined using the method described by Hiai et al. [62]. The other main components of the extract included flavonoids, sugars and phenolic acids [31]. 

### 3.2. Emulsion Preparation

Emulsion samples with a volume of 20 mL were prepared in sterile 50 mL plastic laboratory tubes. The two-step process used [9] had small modifications, such as in the first step where the components were homogenized using a hand homogenizer CAT X120 equipped with a T10 shaft. The samples were mixed at 10,000 rpm for 600 s. In the second step, the samples were homogenized (sonicator Sonoplus, Bandelin, Berlin, Germany) in the following conditions: 10 min, in 10 s/10 s action/break cycles, amplitude of 16%, and cooled with tap water. 

### 3.3. Experimental Design

In this study, a three level Box–Behnken experiment design with response surface methodology (RSM) (Design Expert software version 13.0) was used for determining the nanoemulsion criteria. The variables included the AHE, WPI and HSO concentrations (% *w*/*v*) at three coded levels, i.e., −1, 0, +1. The specific ranges of the variables were selected according to prior knowledge and preliminary studies performed on the formulation of the hempseed oil nanoemulsion. The Box-Behnken design provided 17 experimental trials with five replications for the center point. The optimal formulations for further stability tests and visual observations were chosen based on the droplet size distribution results of the experimental trails.

### 3.4. Methods

#### 3.4.1. Stability Tests: EI Index, UV-Vis, Centrifugation

The emulsion stability was evaluated after 24 h by measuring the proportion of the emulsified phase content to the total volume of the homogenized mixture (EI index). Measurements details were described in [31]. In addition, 5 mL of the samples were taken immediately after homogenization, their optical density at 600 nm (OD_0_) was measured, and then the samples were centrifuged (10,000 RCF, 10 min) and the optical density (OD_C_) was measured again. Evaluation of emulsion stability after centrifugation was determined by the ratio of OD_C_ to OD_0_.

#### 3.4.2. Droplet Size Distribution

Zetasizer Nano-ZS (Malvern, Malvern, UK) was used for measurements of the hydrodynamic diameters (d_H_) of droplets of the emulsion samples. The DLS autocorrelation functions were registered from the scattered light, which was recorded at an angle of 173°. The droplet size distribution measurements were performed with the automatic settings mode at 23.5 °C. Immediately prior to the examinations, the samples were kept at 23.5 °C for 5 min inside the measurement cell (the temperature was adjusted to the average laboratory temperature, where the samples were prepared and stored). Measurement series values and their respective standard deviations were obtained from the average of three measurements.

#### 3.4.3. Microscopic Investigations

The microscopic studies were performed using an inverted microscope ZEISS Axio Vert.A1 (Zeiss, Shanghai, China) equipped with a color camera Axiocam 208 (Zeiss, China). Imaging was performed using two kinds of objectives with different magnifications: LD A-Plan (×40/0.55; phase 1 (air)) and A-Plan (×100/1.25; phase 2 (oil)). For the imaging, the emulsions were inserted into a 1 μ-Slide VI 0.1 cuvette (ibidi GmbH, Gräfelfing, Germany). For the presentation, the resolution of the images was automatically adjusted by the best fit with the ZEN2.5 software (Zeiss, Jena, Germany).

#### 3.4.4. Rheological Tests

A ViscoQC 300 viscometer (Anton Paar GmbH, Graz, Austria) was used to determine the rheological properties of the emulsions. The tests were conducted at room temperature using the “double-gap” DG26 system with an L1 spindle. The speed of the spindle ranged from 7 to 250 rpm, with an increasing trend, which corresponds to a shear rate of 10–322 1/s. Each shear rate was imposed for 1 min to stabilize the viscosity. Each test was performed in triplicate, with fresh samples.

#### 3.4.5. FTIR

The FTIR spectra were obtained using a Spectrum Two FT-IR spectrometer equipped with a Universal ATR with a diamond crystal (PerkinElmer, Waltham, MA, USA). The data were collected over the 4000–500 cm^−1^ spectral range. Typically, a few microliters were used on the diamond, and the measurements were repeated three times for each sample.

#### 3.4.6. Color Analysis

For color evaluation, an NH310 portable spectrophotometer (Shenzhen Threenh Technology Co., Ltd., Shenzhen, China) equipped with internal software was applied. Before the examinations, 2 mL of the sample were inserted into a transparent plastic cuvette. The measurements were carried out in a cuvette inserted into the dedicated measurement chamber. The color tests were repeated 10 times, and the average values with the SD were recorded. The whiteness index (*WI*) of the emulsions was calculated using the following equation [63]:

$$WI=100-{[(100-L*{)}^{2}+a{*}^{2}+b{*}^{2}]}^{1/2}$$

#### 3.4.7. Refractive Index

The refractive indices (RI) were determined using a PAL-RI optical electronic refractometer (ATANGO CO., LTD., Tokyo, Japan). The measurements were repeated 10 times, and the mean value with the SD is given as a final result.

#### 3.4.8. Biological Activity

The bioactivity of the selected emulsions was determined using an environmental cell metabolic activity test of the *Lactobacillus* sp. 2675 strain. The test was performed according to the methodology described by Pacholak et al. (2019). In these assays, 0.25 mL of the emulsion, 0.25 mL of the cell suspension in a nutrient broth (OD_600nm_ ca. 1.0 after 24 h of incubation at 30 °C) and 0.05 mL of the MTT indicator solution (5 mg/mL) were used. After 24 h of incubation, the reduction of yellow MTT into its purple formazan, which is catalyzed by cellular respiratory pathway enzymes, was measured. The samples in which the emulsion was replaced with deionized water were used as the reference sample.

#### 3.4.9. Water Activity

Measurements of water activity of the emulsions were conducted using water diffusion and activity analyzer ADA-7 (COBRABID, Poznan, Poland). The system is equipped with automatic time recording of water evacuation runs from individual samples. Detailed characteristics of the experimental method are specifically described in [64]. The emulsion samples were placed in the measuring vessels. The volume of the sample was 2 mL. The temperature during measurements was stabilized at 20.0 °C ± 0.1 °C using Peltier modules. The chamber was dried to the water activity of 0.05. The duration of one measurement was set to 1400 s.

## Figures and Tables

**Table 1:** Box–Behnken design samples description; AHE—*Aesculus hippocastanum* L. extract; HSO—hempseed oil.

Sample Name	AHE Concentration (%)	Whey Concentration (%)	HSO Concentration (%)	Total Volume with Water (mL)
S07	1	1	3	25
S14	1	2.5	1	
S10	1	2.5	5	
S02	1	4	3	
S04	2	1	1	
S09	2	1	5	
S01	2	2.5	3	
S05	2	2.5	3	
S06 *	2	2.5	3	
S12 *	2	2.5	3	
S15 *	2	2.5	3	
S17	2	4	1	
S08	2	4	5	
S03	3	1	3	
S16	3	2.5	1	
S11	3	2.5	5	
S13	3	4	3	

* Excluded from droplet size distributions tests.

**Table 2:** Preliminary stability tests after centrifugation. OD_C_/OD_0_—ratio of the optical density at 600 nm of samples after and before centrifugation.

Sample	AHE [g/L]	Whey [g/L]	HSO [%]	OD_C_/OD_0_ [-]
1	2	2.5	3	26%
2	1	4	3	44%
3	3	1	3	18%
4	2	1	1	28%
5	2	2.5	3	33%
6	2	2.5	3	30%
7	1	1	3	36%
8	2	4	5	17%
9	2	1	5	41%
10	1	2.5	5	35%
11	3	2.5	5	21%
12	2	2.5	3	33%
13	3	4	3	17%
14	1	2.5	1	31%
15	2	2.5	3	26%
16	3	2.5	1	54%
17	2	4	1	55%

**Table 3:** Preliminary tests, droplet size distribution results: Z-ave (nm); PDI—polydisperisty index; average size distribution by intensity peak maximum (nm) and average size distribution by number peak maximum (nm).

Sample Name	Z-ave (nm)	PDI	Average Size Distribution by Intensity Peak Maximum (nm)	Average Size Distribution by Number Peak Maximum (nm)
S01	229 ± 5	0.344 ± 0.078	369 ± 80	59 ± 31
S02	214 ± 1	0.303 ± 0.022	317 ± 8	30 ± 5
S03	239 ± 7	0.274 ± 0.063	333 ± 44	69 ± 48
S04	234 ± 4	0.182 ± 0.009	279 ± 31	121 ± 39
S05	221 ± 6	0.302 ± 0.010	316 ± 26	27 ± 8
S06	N/A			
S07	215 ± 3	0.291 ± 0.026	286 ± 22	33 ± 5
S08	202 ± 4	0.412 ± 0.016	318 ± 20	23 ± 7
S09	246 ± 2	0.471 ± 0.035	456 ± 46	38 ± 19
S10	195 ± 5	0.439 ± 0.044	306 ± 33	31 ± 19
S11	212 ± 3	0.441 ± 0.029	326 ± 84	74 ± 44
S12	N/A			
S13	218 ± 7	0.322 ± 0.040	322 ± 33	71 ± 46
S14	215 ± 2	0.190 ± 0.018	253 ± 6	129 ± 12
S15	N/A			
S16	232 ± 4	0.183 ± 0.019	288 ± 17	103 ± 42
S17	243 ± 3	0.218 ± 0.038	291 ± 22	98 ± 50

N/A: not applicable; ±SD (standard deviation of three replicates).

**Table 4:** Parameters of the broken power model describing the viscosity curves of the samples: consistency coefficient (K_1_ and K_2_), flow index (n_1_ and n_2_).

Sample Name	Consistency Coefficient K_1_	Flow Behavior Index n_1_	Consistency Coefficient K_2_	Flow Behavior Index n_2_
S02	1.85 ± 0.03 ^a^	0.931 ± 0.002 ^c^	0.093 ± 0.009 ^a^	1.54 ± 0.02 ^b^
S08	2.01 ± 0.04 ^b^	0.923 ± 0.003 ^b^	0.131 ± 0.008 ^c^	1.48 ±0.02 ^a^
S13	1.86 ± 0.02 ^a^	0.934 ± 0.004 ^c^	0.118 ± 0.006 ^b^	1.50 ± 0.01 ^a^
S14	1.85 ± 0.04 ^a^	0.916 ± 0.003 ^a^	0.115 ± 0.007 ^b^	1.50 ± 0.02 ^a^

^a–c^ Values in the same column with the same superscript alphabet letters are not significantly different from each other according to Duncan’s grouping of means.

**Table 5:** Refractive index of the investigated samples.

Sample Name	S02	S08	S13	S14
Refractive index	1.3368	1.3400	1.3384	1.3349

Refractive index accuracy was estimated to be 0.0001.

## Data Availability

All collected data were presented in the manuscript.

## Sample Availability

Samples of HSO are commercially available; samples of AHE and the HSO nanoemulsion might be prepared upon request to the corresponding author.
